# Complete Genome Sequence of *Achromobacter denitrificans* PR1

**DOI:** 10.1128/genomeA.00762-17

**Published:** 2017-08-03

**Authors:** Ana C. Reis, Kevin Kroll, Margarita Gomila, Boris A. Kolvenbach, Philippe F. X. Corvini, Olga C. Nunes

**Affiliations:** a Department of Chemical Engineering, Laboratory for Process Engineering, Environment, Biotechnology and Energy (LEPABE), Faculty of Engineering, University of Porto, Porto, Portugal; b Institute for Ecopreneurship, School of Life Sciences, University of Applied Sciences and Arts Northwestern Switzerland, Muttenz, Switzerland; c Microbiology, Department of Biology, University of the Balearic Islands, Palma de Mallorca, Spain

## Abstract

*Achromobacter denitrificans* strain PR1 was isolated from an enrichment culture able to use sulfamethoxazole as an energy source. Here, we describe the complete genome of this strain sequenced by Illumina MiSeq and Oxford Nanopore MinION.

## GENOME ANNOUNCEMENT

*Achromobacter denitrificans* is a Gram-negative rod-shaped bacterium commonly found in soil and occasionally in human infections (1, 2). Members of this species have previously been linked to the degradation of xenobiotics (3–6), highlighting their potential for bioremediation. Here, we describe the complete genome sequence of *A. denitrificans* strain PR1, originally obtained from enriched activated sludge and able to use sulfamethoxazole (SMX) as an energy source (7).

Strain PR1 was incubated overnight at 30°C in mineral medium B (8) with ammonium sulfate (0.54 g/liter), succinate (0.83 g/liter), yeast extract (0.2 g/liter), and SMX (0.15 g/liter). Genomic DNA extraction was performed with GenElute bacterial genomic DNA kit (Sigma) and sequenced using MiSeq (Illumina) and MinION (Oxford Nanopore). For MiSeq paired-end sequencing (2 × 300 bp), two libraries were independently prepared from 1 µg of DNA with the TruSeq DNA LT sample prep kit (library 1 [lib1]) from Illumina or the Kapa HyperPrep kit (library 2 [lib2]) from Kapa Biosystems. The MinION library was prepared from 1 µg of DNA, sheared into 5-kb fragments with a g-TUBE (Covaris), prepared with the genomic DNA sequencing kit (SQK-MAP-103), and sequenced using a flow cell with R7 chemistry (Oxford Nanopore). The library was loaded in the beginning and after 24 h to coincide with the g1-to-g2 pore switch (9).

MiSeq sequencing generated 2.5 million (lib1) and 0.3 million (lib2) paired-end raw reads. All reads were screened for PhiX contamination and adapter and quality trimmed (>Q20) with the BBDuk tool (https://sourceforge.net/projects/bbmap). MinION sequencing generated 12,591 2D reads (10) (>Q9) that were converted to fastq format with Poretools version 0.5.1 (11). Hybrid *de novo* assembly was done with SPAdes version 3.10.0 (12) with the options -careful and -nanopore. Contigs with <1× coverage were removed from the assembly, resulting in a single scaffold. Circularization was performed with PCR and Sanger sequencing, generating a single circular chromosome of 6,929,205 bp with 46-fold average coverage and 67.4% G+C content.

Analysis with the Rapid Annotations using Subsystems Technology (RAST) server version 2.0 (13) predicted 6,425 protein-coding sequences (CDSs), 4 copies of the rRNA operon, and 59 tRNAs. Functional prediction of the CDSs was further refined by aligning protein sequences against the Gene Ontology (GO) database (14) with InterProScan (15) and BLASTp (16) in Blast2GO version 4.1 (17). Of the total CDSs, 5,210 (81.1%) had a functional prediction, and, from these, 2,939 (45.7%) had catalytic activity (891 hydrolases and 746 oxidoreductases). ResFinder (18) analysis identified multiple antibiotic resistance genes (*sul1*, *sul2*, and *tetC*), with some (*cmlA1h* and *aadA2*) within the new class I integron In1410 (19). Average nucleotide identity (ANI) analysis (20) and *in silico* DNA-DNA hybridization (DDH) analysis (21, 22) with the *A. denitrificans* type strain genome (GenBank accession number BCTQ00000000) showed that strain PR1 belongs to the same species (ANI, 99.33%; DDH, 94.60%; difference in %G+C content, 0.19).

The genome of strain PR1 will provide further insights into sulfamethoxazole metabolism in this microbial consortium and into the species versatility and potential for xenobiotic degradation.

### Accession number(s).

This complete genome sequence has been deposited in GenBank under the accession no. CP020917. The version described in this paper is the first version.
